# Smartphone App for Improving Self-Awareness of Adherence to Edoxaban Treatment in Patients With Atrial Fibrillation (ADHERE-App Trial): Randomized Controlled Trial

**DOI:** 10.2196/65010

**Published:** 2024-11-21

**Authors:** Minjae Yoon, Ji Hyun Lee, In-Cheol Kim, Ju-Hee Lee, Mi-Na Kim, Hack-Lyoung Kim, Sunki Lee, In Jai Kim, Seonghoon Choi, Sung-Ji Park, Taeho Hur, Musarrat Hussain, Sungyoung Lee, Dong-Ju Choi

**Affiliations:** 1 Division of Cardiology, Department of Internal Medicine Seoul National University Bundang Hospital Seoul National University College of Medicine Seongnam Republic of Korea; 2 Division of Cardiology, Department of Internal Medicine Keimyung University Dongsan Hospital Keimyung University School of Medicine Daegu Republic of Korea; 3 Division of Cardiology, Department of Internal Medicine Chungbuk National University Hospital Chungbuk National University College of Medicine Cheongju Republic of Korea; 4 Department of Cardiology Korea University Anam Hospital Korea University College of Medicine Seoul Republic of Korea; 5 Division of Cardiology Seoul National University Boramae Medical Center Seoul Republic of Korea; 6 Department of Cardiology Korea University Guro Hospital Korea University College of Medicine Seoul Republic of Korea; 7 Division of Cardiology, Department of Internal Medicine Bundang CHA Medical Center CHA University Seongnam Republic of Korea; 8 Division of Cardiology, Department of Internal Medicine Kangnam Sacred Heart Hospital Hallym University College of Medicine Seoul Republic of Korea; 9 Division of Cardiology, Department of Internal Medicine Heart Vascular Stroke Institute, Samsung Medical Center Sungkyunkwan University School of Medicine Seoul Republic of Korea; 10 Department of Computer Science and Engineering Kyung Hee University Yongin Republic of Korea; 11 Department of Computer Science UiT The Arctic University of Norway Tromsø Norway

**Keywords:** mobile apps, digital health, atrial fibrillation, anticoagulants, medication adherence, mobile phone

## Abstract

**Background:**

Adherence to oral anticoagulant therapy is essential to prevent ischemic stroke in patients with atrial fibrillation (AF).

**Objective:**

This study aimed to evaluate whether smartphone app–based interventions improve medication adherence in patients with AF.

**Methods:**

This open-label, multicenter randomized controlled trial (ADHERE-App [Self-Awareness of Drug Adherence to Edoxaban Using an Automatic App Feedback System] study) enrolled patients with AF treated with edoxaban for stroke prevention. They were randomly assigned to app-conditioned feedback (intervention; n=248) and conventional treatment (control; n=250) groups. The intervention group received daily alerts via a smartphone app to take edoxaban and measure blood pressure and heart rate at specific times. The control group received only standard, guideline-recommended care. The primary end point was edoxaban adherence, measured by pill count at 3 or 6 months. Medication adherence and the proportion of adequate medication adherence, which was defined as ≥95% of continuous medication adherence, were evaluated.

**Results:**

Medication adherence at 3 or 6 months was not significantly different between the intervention and control groups (median 98%, IQR 95%-100% vs median 98%, IQR 91%-100% at 3 months, *P*=.06; median 98%, IQR 94.5%-100% vs median 97.5%, IQR 92.8%-100% at 6 months, *P*=.15). However, the proportion of adequate medication adherence (≥95%) was significantly higher in the intervention group at both time points (76.8% vs 64.7% at 3 months, *P*=.01; 73.9% vs 61% at 6 months, *P*=.007). Among patients aged >65 years, the intervention group showed a higher medication adherence value and a higher proportion of adequate medication adherence (≥95%) at 6 months.

**Conclusions:**

There was no difference in edoxaban adherence between the groups. However, the proportion of adequate medication adherence was higher in the intervention group, and the benefit of the smartphone app–based intervention on medication adherence was more pronounced among older patients than among younger patients. Given the low adherence to oral anticoagulants, especially among older adults, using a smartphone app may potentially improve medication adherence.

**Trial Registration:**

International Clinical Trials Registry Platform KCT0004754; https://cris.nih.go.kr/cris/search/detailSearch.do?seq=28496&search_page=L

**International Registered Report Identifier (IRRID):**

RR2-10.1136/bmjopen-2021-048777

## Introduction

Stroke prevention with oral anticoagulants is essential in the management of patients with atrial fibrillation (AF) [1,2]. Previous large randomized controlled trials for AF have shown improved safety and comparable efficacy of nonvitamin K antagonist oral anticoagulants (NOACs) compared with warfarin [3]. Thus, current guidelines prefer NOACs over warfarin for ischemic stroke prevention in patients with AF [1,2]. However, with the relatively short half-life of NOACs, when doses are missed, there is a higher risk of a prothrombotic state [4]. Therefore, strict adherence to NOAC therapy is essential to maintain a consistent anticoagulant effect on stroke prevention.

Telemonitoring, feedback, and education result in higher NOAC adherence [5,6]. However, monitoring and feedback by health care professionals require high costs and human resources and, thus, are challenging to incorporate in real clinical practice. With advancements in mobile technology and artificial intelligence, smartphone apps may be a promising strategy to improve medication adherence with ease of use [7]. Although there is some debate about their effectiveness, smartphone apps are currently recognized as an effective method of improving medication adherence [8-14]. Smartphone apps can send push alerts to remind patients to take their medication, which can help improve drug adherence. In addition, active involvement in measuring patients’ health status, including blood pressure (BP) using smartphone apps can improve awareness of their underlying conditions. This, in turn, influences self-care behavior and may increase medication adherence [15].

There have been previous studies on mobile apps for improving adherence to NOACs [16-18]. However, these studies have a small number of participants and limited app functions. Given the low adherence to NOACs in patients with AF in the real world [19-21], we developed a smartphone app that provides push alerts to take NOACs and measure BP and heart rate (HR) at specific times of the day. This study aimed to evaluate whether a smartphone app–based intervention would improve medication adherence compared with usual care in a large population of patients with AF requiring NOACs.

## Methods

### Study Design

The ADHERE-App (Self-Awareness of Drug Adherence to Edoxaban Using an Automatic App Feedback System) study is a prospective, open-label, multicenter randomized controlled trial aimed at evaluating the effect of a smartphone app on improving drug adherence to NOAC in patients with AF. Nine tertiary university hospitals in South Korea participated in this study, which was registered in the International Clinical Trials Registry Platform (KCT0004754).

### Ethical Considerations

This clinical trial was approved by the Institutional Review Boards of Seoul National University Bundang Hospital (B-1904-532-301), Keimyung University Dongsan Hospital, Chungbuk National University Hospital, Korea University Anam Hospital, Seoul National University Boramae Medical Center, Bundang CHA Medical Center, Dongtan Sacred Heart Hospital, Kangnam Sacred Heart Hospital, and Samsung Medical Center. This study was conducted per the Declaration of Helsinki, and all study data were deidentified. All patients provided written informed consent on enrollment.

### Study Population

We enrolled patients with AF aged ≥19 years requiring oral anticoagulation therapy (CHA_2_DS_2_-VASc score ≥1 point for men and ≥2 points for women) for stroke prevention [1]. Patients already taking edoxaban or patients who had switched to edoxaban from other NOACs were enrolled. Edoxaban (Lixiana, Daiichi Sankyo) was administered to patients at an on-label dosage of 60 mg once daily. The dose was reduced to 30 mg once daily in patients who met any of the following criteria: moderate renal impairment (creatinine clearance, 30-50 mL/min), body weight ≤60 kg, or concomitant use of potent P-glycoprotein inhibitors. As the use of smartphones was essential in this study, the participants were required to be familiar with the use of Android smartphones and able to understand and follow Korean instructions for using the app. Patients with severe renal insufficiency (creatinine clearance <15 mL/min), moderate or severe mitral valve stenosis, or a history of mitral valve replacement or repair were excluded. The detailed inclusion and exclusion criteria are listed in Textbox 1.

**Inclusion and exclusion criteria.**

**Inclusion criteria**

1. Patients with atrial fibrillation aged 19 years or older requiring oral anticoagulation therapy (CHA_2_DS_2_-VASc score ≥1 point for men and ≥2 points for women)
2. Patients who are already taking edoxaban or are initiating newly prescribed edoxaban
3. Patients who can use Android smartphones and are fluent in Korean
4. Patients who voluntarily consent to participate in this clinical trial

**Exclusion criteria**

1. Creatinine clearance less than 15 mL/min
2. Patients with dual antiplatelet therapy
3. Moderate or severe mitral stenosis
4. Previous history of mitral valve replacement or mitral valve repair
5. Previous history of alcohol or drug abuse
6. Patients with a mean systolic blood pressure ≥200 mm Hg or diastolic blood pressure ≥110 mm Hg during the screening visit
7. Patients with potential pregnancy or those who are breastfeeding
8. Patients who are judged as both legally and psychologically inadequate to participate in the clinical study by the investigator
9. Patients who have participated in clinical studies with other investigational drug products within 4 weeks before screening
10. Patients unwilling to participate in the clinical study

The CHA_2_DS_2_-VASc score for ischemic stroke risk prediction was calculated by the summation of all assigned points: one point each for congestive heart failure (C), hypertension (H), being aged between 65 and 74 years (A), diabetes mellitus (D), vascular disease (V), and female sex (Sc) and 2 points each for a history of stroke, transient ischemic attack, or thromboembolism (S_2_) or being aged ≥75 years (A_2_) [1,22]. Congestive heart failure was defined as clinical heart failure regardless of ejection fraction, objective evidence of moderate to severe left ventricular dysfunction, or hypertrophic cardiomyopathy. Vascular disease was defined as angiographically significant coronary artery disease, previous myocardial infarction, peripheral vascular disease, or aortic plaques.

### Patient Recruitment and Randomization

Any patient who presented to the clinic for AF management was considered a potential participant. The research nurses conducted an initial screening of the patients’ eligibility criteria and access to a smartphone. Of these patients, after a final comprehensive screening and interview, written informed consent was obtained from eligible participants. After enrollment, the participants were randomized 1:1 to either the intervention group (app-conditioned feedback group) or the control group (conventional treatment group) using a web-based central randomization service [23]. Randomization was performed by research nurses using permuted block randomization derived from SAS (version 9.2, SAS Institute). Owing to the nature of the intervention, this study’s participants and investigators interacting with the patients were not blinded to the group allocation. All the participants were informed that information on edoxaban adherence would be collected for analysis.

### Intervention (Smartphone App–Conditioned Feedback) and Follow-Up

We developed a smartphone app that provides push alerts to take edoxaban and measure BP and HR at specific times of the day. After randomization, this study’s participants in the intervention group installed this study’s app provided by the research nurses on their Android smartphones. As it was not an open app accessible to the public, the control group could not access it. The operating system and display of the smartphone app are described in a previously published design paper [24] and in Multimedia Appendix 1.

In brief, the research nurses initially set an alarm for taking edoxaban at a certain time of the day in this study’s app, and the participants were able to change the time of the alarm. When the alarm was heard at the preset time, the patient was prompted to take edoxaban, and the app also checked whether the patient took their medication. In addition to the alarm for taking medication, the app sent an alarm to measure BP and HR using an automated BP monitor (UA-651BLE, A&D Medical). The measured BP and HR data were automatically transmitted to a smartphone via Bluetooth. To assess awareness of disease status, the app asked patients whether their measured BP results were optimal, and the patients were required to answer the question. The patients could review their historical vital sign data entered by date using the smartphone app. Physicians could obtain the patient’s BP and HR readings by checking the patient’s smartphone app at the follow-up visit or they could monitor the patient’s vital signs in the standalone web dashboard system.

The control group received only standard, guideline-recommended care [1,2], including education about the disease and the importance of taking medication. The control group received the same automated BP monitor as the intervention group but did not have access to the smartphone app. Patients in both the intervention and control groups were followed up at 3 and 6 months after randomization. During this study, patients needed to continue taking edoxaban unless there were special circumstances that required an interruption or change. Discontinuation of edoxaban could be considered if creatinine clearance drops below 15 mL/min or in case of major bleeding or other serious events during this study.

### Study Outcomes

The primary end point of this study was adherence to edoxaban at 3 and 6 months. Medication adherence was evaluated with the “pill count” measurements. The patients brought the remaining tablets to each scheduled visit, and trained research nurses counted the number of returned drugs and calculated medication adherence as follows:



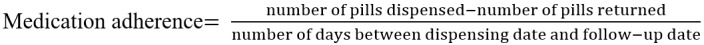



Medication adherence at 3 and 6 months was defined as the value from baseline to 3 months and from baseline to 6 months, respectively. We also defined another primary end point, adequate medication adherence, as ≥95%, taking into account previous studies [25-27], and compared the proportion of adequate medication adherence (≥95%) between the intervention and control groups. Medication adherence was analyzed as a continuous variable, while adequate medication adherence was treated as a binary variable. Accordingly, we examined both the actual adherence values and the proportion of patients with adequate adherence.

The secondary end points were clinical composite end points, including stroke, systemic embolic events, major bleeding requiring hospitalization or transfusion, and death during the 6-month follow-up period. The exploratory clinical outcome was the frequency of app use during the 6 months, as assessed by the input of vital signs into the app.

### Sample Size and Statistical Analyses

To the best of our knowledge, no large studies have evaluated the use of a smartphone app to improve NOAC adherence using pill count measurements. Thus, a precise sample size calculation was not possible. Considering previous studies, drug adherence was assumed to be 90% in the control group and 95% in the intervention group with a SD of 15%, considering previous studies [16,28]. With a 2-tailed α of .05, a power of 0.95, and a dropout rate of 6%, 500 patients (250 patients in each group) were required.

Categorical variables are reported as frequencies (percentages) and were compared using the Pearson chi-square test or Fisher exact test. Continuous variables were tested for normality using the Shapiro-Wilk normality test. Continuous variables with normal distribution are expressed as means (SDs) and were compared using the 2-tailed Student *t* test. Nonnormally distributed continuous variables are presented as medians with IQRs and were compared using the Mann-Whitney *U* test.

Analyses were performed according to the intention-to-treat analysis principle and included data from all patients who had undergone valid randomization. For patients who discontinued the trial early, we used the data available before discontinuation of the trial to calculate medication adherence at 3 or 6 months. If medication adherence could not be verified by pill count measurements or any other reliable method, the data were considered missing and excluded from the primary analysis. In the subgroup analyses, we evaluated the differential effects of the intervention on primary outcomes concerning sex and age. To assess the feasibility and effectiveness of the smartphone app function, an additional analysis was performed on the intervention group to determine whether adherence to edoxaban differed according to the frequency of app use.

All tests were 2-tailed, and a *P* value <.05 was considered statistically significant. Statistical analyses were performed using R (version 4.2.3, R Foundation).

## Results

### Patient Enrollment and Clinical Characteristics

**Figure 1:**
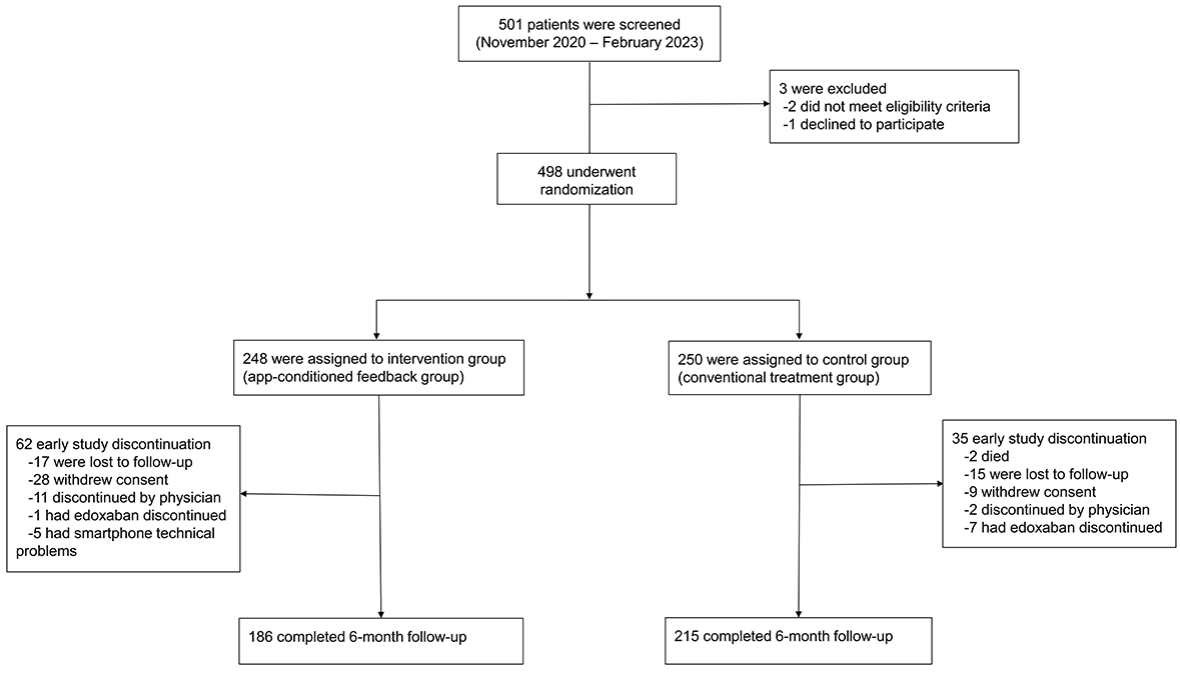
CONSORT (Consolidated Standards of Reporting Trials) flow diagram.

From November 2020 to February 2023, a total of 501 patients with AF were screened for eligibility, of whom 498 were randomly assigned to the intervention (n=248) or control (n=250) group (Figure 1 and the CONSORT [Consolidated Standards of Reporting Trials] checklist in Multimedia Appendix 2). The demographic and clinical characteristics of the 2 groups were well balanced at baseline (Table 1). The mean age was 65.7 years, and 68.3% (340/498) were male. The median CHA_2_DS_2_-VASc score was not significantly different between the two groups (median 3, IQR 2-3 vs median 2, IQR 2-3; *P*=.66). The mean systolic BP, diastolic BP, and HR of the total population were 126.7 mm Hg, 78.5 mm Hg, and 75.6 beats per minute, respectively.

The diagram indicates the number of patients screened, enrolled, and randomized in the trial and this study’s flow.

End-of-study visits occurred on September 12, 2023, which was the final date of the follow-up data. Among the 498 study participants, 401 (80.5%) completed the 6-month follow-up, and 97 patients discontinued follow-up in the trial before study completion (62 in the intervention group and 35 in the control group).

**Table 1:** Baseline characteristics according to treatment groups.

Variables	Intervention (n=248)	Control (n=250)	*P* value
Age (years), mean (SD)	65.7 (10.1)	65.6 (10.5)	.89
Male, n (%)	171 (69)	169 (67.6)	.82
Height (cm), mean (SD)	165.2 (9.3)	166.1 (8.8)	.28
Weight (kg), mean (SD)	69.3 (12.8)	69.8 (12.1)	.67
Alcohol, n (%)	84 (33.9)	81 (32.4)	.80
Smoking, n (%)	28 (11.3)	35 (14)	.44
Heart failure, n (%)	115 (46.4)	119 (47.6)	.85
Hypertension, n (%)	145 (58.5)	138 (55.2)	.52
Diabetes mellitus, n (%)	60 (24.2)	69 (27.7)	.44
Previous stroke, transient ischemic attack, or thromboembolism, n (%)	13 (5.2)	16 (6.4)	.72
Vascular disease, n (%)	48 (19.4)	42 (16.8)	.75
CHA_2_DS_2_-VASc score, median (IQR)	3 (2-3)	2 (2-3)	.66
Systolic blood pressure (mm Hg), mean (SD)	126.3 (18.2)	127.1 (17.4)	.62
Diastolic blood pressure (mm Hg), mean (SD)	78.2 (12.1)	78.8 (11.9)	.61
Heart rate (beat per minute), mean (SD)	75 (13.8)	76.1 (15.4)	.41

### Medication Adherence According to Treatment Groups

**Figure 2:**
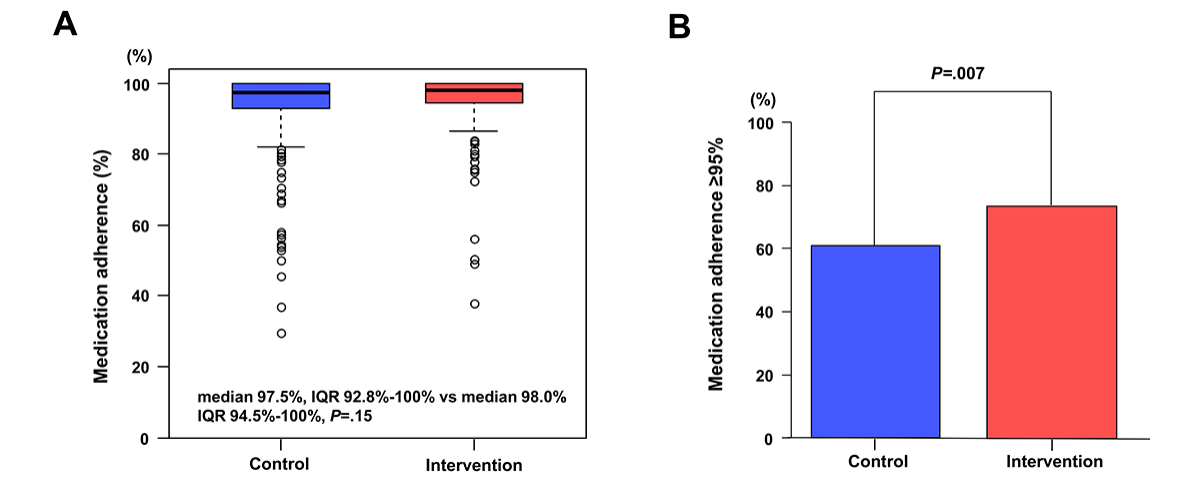
Medication adherence according to treatment groups. (A) Medication adherence at 6 months and (B) proportion of patients achieving adequate medication adherence (≥95%) at 6 months.

Medication adherence according to treatment group and primary outcome is presented in Table 2. Medication adherence to edoxaban at 3 or 6 months was assessed in 405 patients (190 in the intervention group and 215 in the control group) and 422 patients (199 in the intervention group and 223 in the control group), respectively. Medication adherence at 3 or 6 months was not significantly different between the intervention and control groups (median 98%, IQR 95%-100% vs median 98%, IQR 91%-100% at 3 months, *P*=.06; median 98%, IQR 94.5%-100% vs median 97.5%, IQR 92.8%-100% at 3 months, *P*=.15; Figure 2). However, the proportion of adequate medication adherence (≥95%) was significantly higher in the intervention group than in the control group at both 3 and 6 months (146/190, 76.8% vs 139/215, 64.7% at 3 months, *P*=.01; 147/199, 73.9% vs 136/223, 61% at 3 months, *P*=.007).

**Table 2:** Medication adherence according to treatment groups.

Variables	Intervention (n=248)	Control (n=250)	Odds ratio (95% CI)^a^	*P* value^b^
Medication adherence at 3 months (%), median (IQR)^c^	98 (95-100)	98 (91-100)	—^d^	.06
The proportion of adequate medication adherence (≥95%) at 3 months, n/N (%)^e^	146/190 (76.8)	139/215 (64.7)	1.81 (1.17-2.81)	.01
Medication adherence at 6 months (%), median (IQR)	98 (94.5-100)	97.5 (92.8-100)	—	.15
The proportion of adequate medication adherence (≥95%) at 6 months, n/N (%)	147/199 (73.9)	136/223 (61)	1.8 (1.2-2.74)	.007

^a^Odds ratio for categorical variables.

^b^*P* value was calculated using the chi-square test for categorical variables and the Mann-Whitney *U* test for continuous variables.

^c^Medication adherence at 3 months was assessed in 405 patients (190 in the intervention group and 215 in the control group).

^d^Not available.

^e^Medication adherence at 6 months was assessed in 422 patients (199 in the intervention group and 223 in the control group).

### Subgroup Analysis of the Primary End Point

Table 3 shows subgroup analyses regarding the difference in medication adherence at 6 months according to the intervention and control groups. When stratified by sex, medication adherence at 6 months was not significantly different between the two groups for either male or female (median 98%, IQR 94.5%-100 vs median 97.5%, IQR 93%-100%, *P*=.42 for male; median 98.5%, IQR 94.5%-100% vs median 96.8%, IQR 92.5%-100%, *P*=.18 for female). However, among patients aged >65 years, the intervention group showed higher medication adherence at 6 months than the control group (median 98%, IQR 96%-100% vs median 97%, IQR 93%-100%; *P*=.03). Moreover, the proportion of adequate drug adherence (≥95%) was significantly higher in the intervention group than in the control group (82/101, 81.2% vs 66/112, 58.9%; *P*=.001). In patients aged ≤65 years, there was no significant difference between the 2 groups per medication adherence (*P*=.92) and the proportion of adequately adherent patients (*P*=.73).

**Table 3:** Subgroup analyses of the difference between the intervention and control groups regarding medication adherence at 6 months^a^.

Variables		Intervention	Control	*P* value
**Male (n=288)**				
	Medication adherence at 6 months (%), median (IQR)	98 (94.5-100)	97.5 (93-100)	.42
	The proportion of adequate medication adherence (≥95%) at 6 months, n/N (%)	102/139 (73.4)	95/149 (63.8)	.10
**Female (n=134)**				
	Medication adherence at 6 months (%), median (IQR)	98.5 (94.5-100)	96.8 (92.5-100)	.18
	The proportion of adequate medication adherence (≥95%) at 6 months, n/N (%)	45/60 (75)	41/74 (55.4)	.03
**Age >65 y (n=213)**				
	Medication adherence at 6 months (%), median (IQR)	98 (96-100)	97 (93-100)	.03
	The proportion of adequate medication adherence (≥95%) at 6 months, n/N (%)	82/101 (81.2)	66/112 (58.9)	.001
**Age ≤65 y (n=209)**				
	Medication adherence at 6 months (%), median (IQR)	97.8 (92.5-100)	97.5 (92-100)	.92
	The proportion of adequate medication adherence (≥95%) at 6 months, n/N (%)	65/98 (66.3)	70/111 (63.1)	.73

^a^Medication adherence at 6 months was assessed in 422 patients (199 in the intervention group and 223 in the control group).

### Secondary End Points and Application Frequency

During the 6-month period, one participant in the control group experienced an ischemic stroke, and one participant each in the intervention and control groups experienced major bleeding. Two patients in the control group died during follow-up. The composite clinical end points were not significantly different between the two groups. No adverse effects were associated with the use of the smartphone app during the trial.

Of the 186 patients in the intervention group who completed 6 months of follow-up, the median frequency of app use during this period, as assessed by the input of vital signs into the app, was 65.6% (IQR 16.7%-91.7%), and 107 (57.5%) patients had a frequency of app use of ≥50%. When patients were categorized into tertiles based on the frequency of app use, they were further divided into groups with values of <30% (n=60), between 30% and 80% (n=58), and >80% (n=68). There was no significant difference in medication adherence to edoxaban at 6 months between the 3 groups (median 98.5%, IQR 95.5%-100% vs median 97.5%, IQR 93%-100% vs median 98%, IQR 95.5%-100%; *P*=.11). The proportion of adequate medication adherence (≥95%) was not significantly different among the 3 groups (45/60, 75% vs 42/58, 72.4% vs 5568, 80.9%; *P*=.51), although the highest tertile of the frequency of app use showed a higher trend toward adequate medication adherence.

## Discussion

### Principal Findings

We developed a mobile app to provide NOAC alerts and measure vital signs. The main findings of this study are as follows: (1) contrary to our expectation, there were no significant differences in medication adherence to edoxaban between the app-based intervention and conventional treatment groups; (2) however, this smartphone app-based intervention resulted in a higher proportion of adequate medication adherence (≥95%) than the conventional treatment; and (3) moreover, in patients aged >65 years, the intervention group showed a higher medication adherence value and a higher proportion of adequate medication adherence at 6 months than the control group.

### Comparison to Prior Work

Poor drug adherence to NOACs can increase the risk of ischemic stroke, leading to increased overall health care costs [19,29]. Therefore, consistent drug adherence to NOAC therapy is important for reducing the risk of ischemic stroke. Interventions such as mobile phone text messaging, telemonitoring, feedback, and education result in higher adherence to NOACs [5,6,30]. However, these interventions by health care providers require time and financial and human resources and are therefore challenging to implement in a real-world clinical setting. With the advent of mobile technology and artificial intelligence, mobile-based reminders or feedback may be a promising strategy to improve medication adherence in chronic diseases, including AF, using cost-effective and accessible methods [8]. We believe that our approach of using a smartphone app–based intervention would be an efficient way to improve NOAC adherence in AF with minimal cost and ease of use.

There have been some previous studies on mobile apps for improving drug adherence to NOACs [16-18]. However, the studies by Labovitz et al [16] and Turakhia et al [17] were limited by a small number of patients (28 and 139 patients, respectively). The study by Turakhia et al [17] did not show a difference in medication adherence following smartphone intervention; however, their smartphone app functions were different from those of ours, as our mobile app functions mainly focused on medication reminders and vital sign measurements. Recently, another study demonstrated that intake reminders increased adherence to NOACs in patients with stroke attributable to AF [31]. We believe that these heterogeneous and diverse functions of mobile interventions and different frequencies of app use by participants can affect the benefits and outcomes of mobile apps, particularly for improving medication adherence.

Adherence to NOACs in clinical trials tends to be higher than that in the real world, which may be because patients feel monitored and measured [5,6,19-21]. In this trial, there was no statistically significant difference in the continuous value of adherence to NOACs between the intervention and control groups. This finding may be because both groups had higher levels of adherence than initially expected. However, the proportion of adequate medication adherence (≥95%), which may be the more important aspect than medication adherence value, was significantly higher in the intervention group than in the control group, demonstrating the benefit of the smartphone app–based intervention in improving medication adherence.

One of the interesting findings of this study was that smartphone-based intervention increased medication adherence to NOACs, particularly in older patients (>65 years). In general, older patients have lower medication adherence than younger patients for several reasons, particularly cognitive problems, which can cause them to forget to take their medication [32]. Specifically, in our study, the proportion of adequate medication adherence in the control group among patients aged >65 years was 58.9%, which is relatively low compared with those of the other subgroups. However, the benefit of smartphone app-based interventions was more pronounced in older patients than in younger patients, with 81.2% demonstrating adequate medication adherence. Therefore, the potential to improve medication adherence in older patients using smartphone apps is of great clinical importance.

The aim of this study differed from that of the ongoing RIVOX-AF (Reinforcement of Adherence via Self-Monitoring App Orchestrating Biosignals and Medication of RivaroXaban in Patients With Atrial Fibrillation and Co-Morbidities) study [33] at our institution. The RIVOX-AF trial aimed to evaluate whether a smartphone app-based intervention, including an alert for drug intake, visual confirmation of medication administration, and a list of medication intake histories, would increase drug adherence. In the ADHERE-App study, patients received a reminder to take their medication and measure their HR and BP according to a prespecified schedule. The RIVOX-AF and ADHERE-App studies have similar app functions for medication intake reminders; however, the ADHERE-App study was designed for active participation in vital sign measurement with the idea that using a smartphone app–based feedback system could help patients become more aware of their underlying conditions, which in turn could influence their self-care habits and improve medication adherence. The RIVOX-AF study mainly focused on confirming medication intake using a camera check.

### Limitations and Strengths

This study has several limitations. First, actual engagement with the apps and actual participant responses to reminders or feedback from the app could not be analyzed. Additionally, the frequency of app use was evaluated based on the input of vital signs, which may not accurately reflect the actual use of the app. These limitations are common in studies using mobile apps. Second, in NOAC studies, it is important to determine whether more than one dose was skipped or an “unprotected day” [34], which was not assessed in this study. Third, an electronic Medication Event Monitoring System using a special cap that fits on a medication bottle to record the exact date and time of bottle opening is one of the best methods for measuring medication adherence and has been used in several other trials [5,6,25]; however, it was not used in this trial. However, pill count measurement by a clinical research nurse is also a relatively accurate assessment of medication adherence and may be more accurate than the drug score [35,36]. Fourth, data on satisfaction with the app or quality of life were not examined. Fifth, there was a significant difference between the intervention and control groups in the number of patients who dropped out or withdrew consent before the end of our study, suggesting that the use of smartphone apps may be associated with a high dropout rate. Sixth, additional methods to adjust for type I error inflation were not considered in the subgroup analyses. Therefore, results from subgroup analyses are exploratory and should be interpreted with caution, even for the subgroup (ie, age >65 years) that differs between the intervention and control groups. In addition, the proportion of adequate medication adherence was added as a coprimary end point after this study was first planned.

Despite these limitations, this study is a nationwide, multicenter randomized control trial with a relatively large number of participants compared with previous studies [16,17]. The major strength of this study is the use of a mobile app with various functions, and we believe that the features of our app can be effective solutions for improving medication adherence in patients with AF.

### Conclusions

There is no difference in adherence to edoxaban between the app-conditioned feedback and conventional treatment groups. However, the proportion of patients with adequate adherence is higher in the intervention group than in the control group, and the benefit of the smartphone app–based intervention on medication adherence is more pronounced among older patients than among younger patients. Given the low adherence to NOACs, especially among older adults, a smartphone app has the potential to improve medication adherence.

## Appendix

Multimedia Appendix 1 Operating system and sample display of the smartphone app.

Multimedia Appendix 2 CONSORT-eHEALTH checklist (V 1.6.1).

## Abbreviations

ADHERE-App: Self-Awareness of Drug Adherence to Edoxaban Using an Automatic App Feedback System
AF: atrial fibrillation
BP: blood pressure
CONSORT: Consolidated Standards of Reporting Trials
HR: heart rate
NOAC: nonvitamin K antagonist oral anticoagulant
RIVOX-AF: Reinforcement of Adherence via Self-Monitoring App Orchestrating Biosignals and Medication of RivaroXaban in Patients With Atrial Fibrillation and Co-Morbidities

## Data Availability

The data generated in this study are available from the corresponding author on reasonable request.
