# Supplementary material for: Smartphone App for Improving Self-Awareness of Adherence to Edoxaban Treatment in Patients With Atrial Fibrillation (ADHERE-App Trial): Randomized Controlled Trial
Source: J Med Internet Res. 2024 Nov 21;26:e65010. doi: 10.2196/65010 (PMC11621717; doi:10.2196/65010)
Supplement: Multimedia Appendix 1 [file jmir_v26i1e65010_app1.docx]

**Multimedia Appendix 1.** Operating system and sample display of the smartphone app.


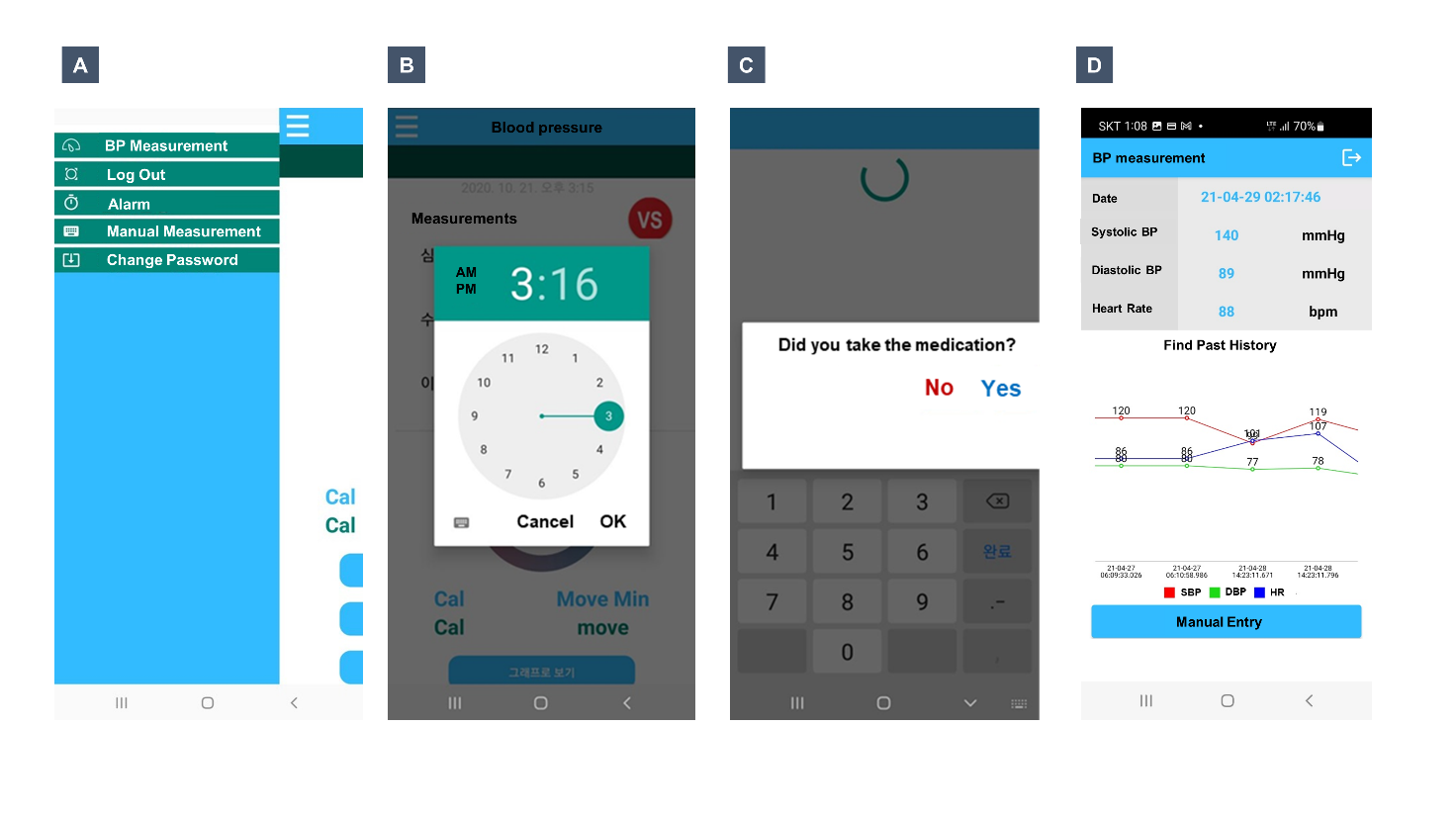


(A) Menu bar showing BP measurement, log out, alarm setting, manual BP input, and password setting; (B) alarm setting page for medication; (C) alarm page to check whether the patient has taken medication; (D) page displaying BP and heart rate measurements.

BP, blood pressure.
